# Visualization of basement membranes by a nidogen-based fluorescent reporter in mice

**DOI:** 10.1016/j.mbplus.2023.100133

**Published:** 2023-04-08

**Authors:** Sugiko Futaki, Ayano Horimoto, Chisei Shimono, Naoko Norioka, Yukimasa Taniguchi, Hitomi Hamaoka, Mari Kaneko, Mayo Shigeta, Takaya Abe, Kiyotoshi Sekiguchi, Yoichi Kondo

**Affiliations:** a Department of Anatomy and Cell Biology, Faculty of Medicine, Osaka Medical and Pharmaceutical University, 2-7 Daigaku-machi, Takatsuki, Osaka 569-8686, Japan; b Laboratory of Matrixome Research and Application, Institute for Protein Research, Osaka University, 3-2 Yamada-oka, Suita, Osaka 565-0871, Japan; c Laboratory for Animal Resources and Genetic Engineering, RIKEN Center for Biosystems Dynamics Research, 2-2-3 Minatojima-minamimachi, Chuo-ku, Kobe, Hyogo 650-0047, Japan

**Keywords:** Basement membrane, Nidogen-1, Live imaging, Reporter mouse, Retina, Vascular basement membrane

## Abstract

Basement membranes (BMs) are thin, sheet-like extracellular structures that cover the basal side of epithelial and endothelial tissues and provide structural and functional support to adjacent cell layers. The molecular structure of BMs is a fine meshwork that incorporates specialized extracellular matrix proteins. Recently, live visualization of BMs in invertebrates demonstrated that their structure is flexible and dynamically rearranged during cell differentiation and organogenesis. However, the BM dynamics in mammalian tissues remain to be elucidated. We developed a mammalian BM imaging probe based on nidogen-1, a major BM-specific protein. Recombinant human nidogen-1 fused with an enhanced green fluorescent protein (Nid1-EGFP) retains its ability to bind to other BM proteins, such as laminin, type IV collagen, and perlecan, in a solid-phase binding assay. When added to the culture medium of embryoid bodies derived from mouse ES cells, recombinant Nid1-EGFP accumulated in the BM zone of embryoid bodies, and BMs were visualized *in vitro*. For *in vivo* BM imaging, a knock-in reporter mouse line expressing human nidogen-1 fused to the red fluorescent protein mCherry (R26-CAG-Nid1-mCherry) was generated. R26-CAG-Nid1-mCherry showed fluorescently labeled BMs in early embryos and adult tissues, such as the epidermis, intestine, and skeletal muscles, whereas BM fluorescence was unclear in several other tissues, such as the lung and heart. In the retina, Nid1-mCherry fluorescence visualized the BMs of vascular endothelium and pericytes. In the developing retina, Nid1-mCherry fluorescence labeled the BM of the major central vessels; however, the BM fluorescence were hardly observed in the peripheral growing tips of the vascular network, despite the presence of endothelial BM. Time-lapse observation of the retinal vascular BM after photobleaching revealed gradual recovery of Nid1-mCherry fluorescence, suggesting the turnover of BM components in developing retinal blood vessels. To the best of our knowledge, this is the first demonstration of *in vivo* BM imaging using a genetically engineered mammalian model. Although R26-CAG-Nid1-mCherry has some limitations as an *in vivo* BM imaging model, it has potential applications in the study of BM dynamics during mammalian embryogenesis, tissue regeneration, and pathogenesis.

## Highlights

• Established a mouse model for the basement membrane live imaging.
• Recombinant nidogen-1, tagged with a fluorescent protein EGFP or mCherry, labeled the basement membranes both *in vitro* and *in vivo*.
• The nidogen-based fluorescent probes enable monitoring the dynamics of basement membranes in mouse tissues/organoids.

## Introduction

Basement membranes (BMs) are thin, sheet-like extracellular structures that cover the basal side of the epithelial and endothelial cell layers. Several mesenchymal cells, such as adipocytes, pericytes, Schwann cells, and muscle cells, are also surrounded by BMs. BMs provide structural and physiological scaffolds for these cells via cell adhesion receptors. BMs are composed of specialized extracellular matrix (ECM) molecules, such as laminin, type IV collagen, perlecan, and nidogen. Laminin and type IV collagen self-polymerize independently to form fine networks that provide a structural basis for BMs, whereas perlecan and nidogen bind to and cross-link these networks to provide structural support and endow functional variation to BMs [1], [2].

The formation of BMs is critical for embryonic cell survival, migration, and differentiation (3), and the molecular composition of BMs varies according to the tissue type and developmental stage [4], [5]. In adult tissues, aberrations in BMs are closely associated with pathological changes, such as cancer growth and metastasis [3], [6]. Histological and developmental studies have implied that BMs are flexible and dynamic structures, particularly during embryogenesis [7], [8], [9]. However, the details of BM dynamics are largely unknown.

Several trials have reported *in vivo* visualization of BMs. For example, the injection of a fluorescent-labeled antibody against laminin delineated BM remodeling during the budding of Hydra [10]. Various BM-imaging models have been studied in Drosophila and Caenorhabditis elegans by genetically labeling BM-specific proteins. Observation of a fluorescently tagged BM protein, laminin-GFP, in Caenorhabditis elegans revealed that breach of the BM by anchor cells causes BM sliding during uterine-vulva attachment [11]. Similarly, GFP-viking (a homolog of mammalian type IV collagen) in Drosophila delineates BM remodeling during the evagination of wing imaginal disks [12], [13]. In mammalian tissues, the BM morphology and dynamics in explant cultures of mouse embryonic submandibular glands have been studied using fluorescently tagged BM-targeting antibodies [14]. However, *in vivo* labeling of BMs, as demonstrated in Drosophila and Caenorhabditis elegans, has not yet been reported in vertebrates.

In this study, we used recombinant nidogen-1 as a probe to visualize the BMs. Two nidogen proteins, nidogen-1 and −2, are encoded in human and mouse genomes. Nidogen-1 (also known as entactin) is a constitutive BM protein ubiquitously expressed in almost all BMs [15]. This approximately 150 kDa glycoprotein consists of three globular (LG) domains, which bind to and cross-link laminin, type IV collagen, and perlecan through its two N-terminal LG domains [15], [16], [17], [18]. Loss of nidogen-1 in mice did not result in any abnormalities, possibly because of the functional redundancy of nidogen-2 [19], [20], [21]. Because nidogen-1 is a secreted monomeric protein with high and specific binding affinities to laminin and other BM molecules, we generated a fluorescent protein-tagged nidogen-1 expression system to label BMs. The addition of the fluorescent protein EGFP or mCherry at the C-terminus of nidogen-1 (Nid1-EGFP or Nid1-mCherry, respectively) did not affect its function *in vitro*. We generated a ROSA26 knock-in reporter mouse line expressing Nid1-mCherry (R26-CAG-Nid1-mCherry) to examine BM labeling *in vivo* [22], [23].

R26-CAG-Nid1-mCherry mice showed mCherry fluorescence in the BMs of multiple tissues at both the developmental and adult stages. To the best of our knowledge, this is the first report of a genetically labeled BM imaging model in vertebrates. In this study, we characterized fluorescent protein-tagged Nid1 as a BM-labeling probe and evaluated its efficiency in R26-CAG-Nid1-mCherry mice.

## Results

### Expression and evaluation of recombinant Nid1-EGFP

**Fig. 1:**
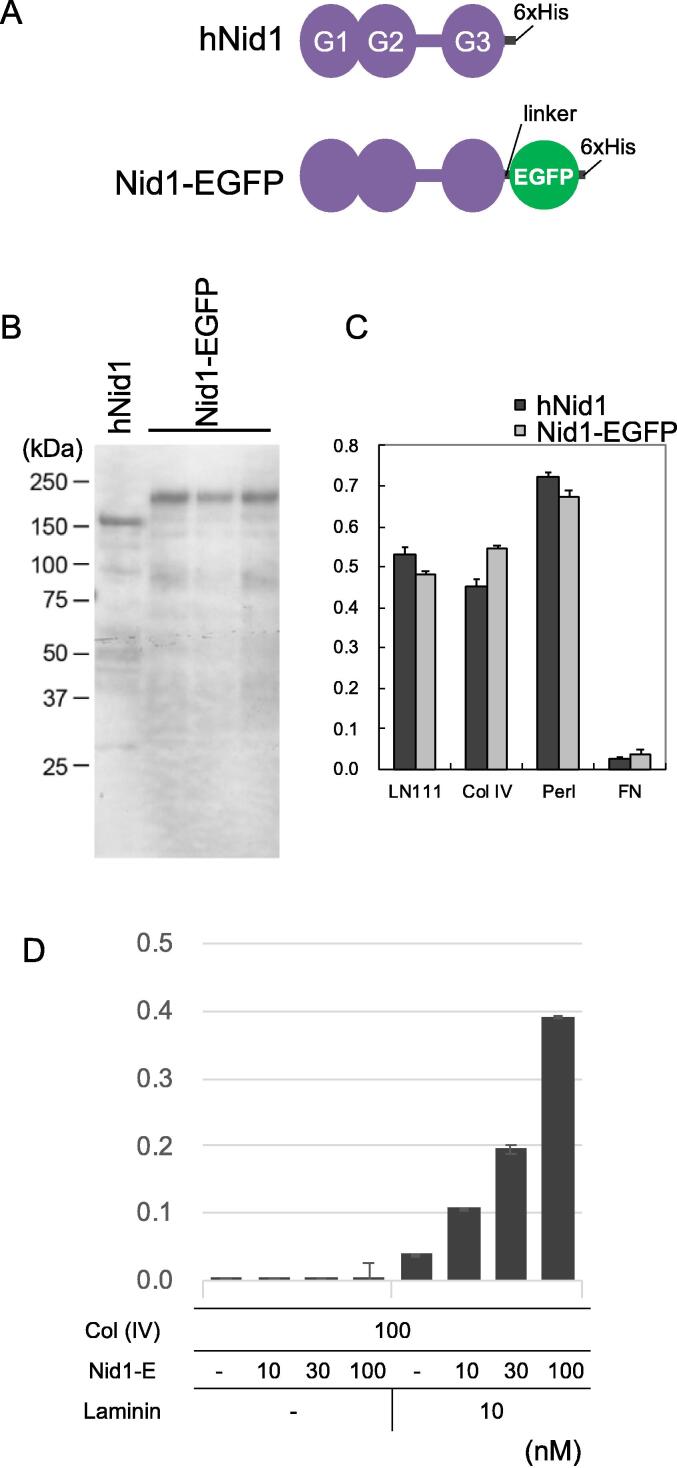
Scheme of recombinant hNid1 and Nid1-EGFP. A) Nidogen-1 (hNid1) protein consists of three globular domains G1, G2, and G3, with a “rod” region between G2 and G3. Nid1-EGFP comprises hNid1, a short linker sequence of four amino acids, EGFP, and six histidine residues (6xHis) at the C-terminus of hNid1. B) Western blot of purified recombinant hNid1 and Nid1-EGFP. Recombinant hNid1 and Nid1-EGFP were transiently expressed in 293F cells and affinity-purified from the conditioned media. Purified proteins were subjected to sodium dodecyl sulfate–polyacrylamide gel electrophoresis (SDS-PAGE) under reducing conditions and detected by western blotting using an anti-His-tag antibody. Three batches of Nid1-EGFP proteins were compared with a batch of hNid1 protein. The predicted molecular weights of hNid1 and Nid1-EGFP were 138 and 165 kDa, respectively. C) Binding of hNid1 and Nid1-EGFP to other BM proteins. In the solid-phase binding assay, purified hNid1 and Nid1-EGFP were incubated with laminin-111 (LN111), type IV collagen (Col IV), perlecan (Perl), and fibronectin (FN) coated on 96-well plates. The vertical axis represents absorbance at 490 nm, indicating the amount of hNid1 and Nid1-EGFP bound to the plate coated with ECM proteins. Each value and error bar represent the mean of three independent assays and the standard deviation, respectively. D) A two-step solid-phase binding assay demonstrated the simultaneous dual-binding ability of Nid1-EGFP to type IV collagen and laminin. Type IV collagen (Col (IV)) was coated onto a 96-well plate at a concentration of 100 nM. Purified Nid1-EGFP (Nid1-E) at concentrations of 10, 30, and 100 nM was overlaid on coated type IV collagen. After washing out the excess Nid1-EGFP, 10 nM FLAG-tagged laminin-111 (laminin) was overlaid on the plate and detected using an anti-FLAG primary antibody and HRP-conjugated secondary antibody. The vertical axis represents the absorbance at 490 nm, which indicates the amount of FLAG-tagged laminin-111 captured on the plate. Each value and error bar represent the mean of three independent assays and the standard deviation, respectively.

Recombinant human nidogen-1 (hNid1) was used to target the BMs. cDNAs encoding hNid1 with six histidine residues (His-tag) and hNid1 fused with EGFP and His-tag (Nid1-EGFP) (Fig. 1A) were expressed in 293F cells. Recombinant hNid1 and Nid1-EGFP were purified from the conditioned media and analyzed using western blotting (Fig. 1B). With predicted molecular weights of 138 kDa for hNid1 and 165 kDa for Nid1-EGFP, each of the three batches of purified Nid1-EGFP showed a band higher than that of hNid1, which was approximately 150 kDa (Fig. 1B).

The binding of hNid1 and Nid1-EGFP to other ECM proteins was examined using a solid-phase binding assay. Both hNid1 and Nid1-EGFP showed high binding affinities to laminin, type IV collagen, and perlecan but not to fibronectin (Fig. 1C), indicating that recombinant Nid1-EGFP and hNid1 retained high selective binding activities to BM proteins. Furthermore, using a two-step solid-phase binding assay, we confirmed that recombinant Nid1-EGFP could simultaneously bind to type IV collagen and laminin (Fig. 1D), suggesting that recombinant Nid1-EGFP retained the ability to act as a crosslinker for BM assembly.

## Labeling of BMs *in vitro* with recombinant Nid1-EGFP

**Fig. 2:**
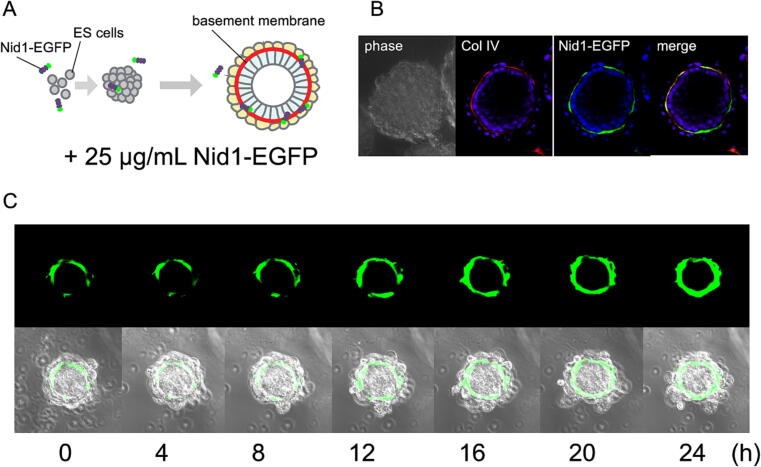
Incorporation of Nid1-EGFP into the BM zone of embryoid bodies *in vitro*. A) Experimental diagram for embryoid body differentiation. Mouse ES cells were cultured in suspension for seven days to differentiate into cystic embryoid bodies in the presence of 25 µg/ml purified recombinant Nid1-EGFP in the culture media. B) A confocal image of Nid1-EGFP in the BM zone of an embryoid body. The embryoid bodies were fixed and immunostained for type IV collagen (Col IV) to visualize the BM. Phase-contrast image (phase), Nid1-EGFP derived fluorescence (Nid1-EGFP), and merged image (merge) are indicated. Nuclei were stained with DAPI and shown in blue in the fluorescent panels. Each confocal image represents a single optical plane. C) Time-lapse imaging of Nid1-EGFP incorporation in the embryoid body. A single embryoid body at day 7 with Nid1-EGFP was observed under confocal microscopy and time-lapse recording. Upper panels show Nid1-EGFP fluorescence in a single optical plane at each time point, and lower panels are those merged with phase-contrast images. Numbers indicate hours (h) after the start of time-lapse imaging. (For interpretation of the references to color in this figure legend, the reader is referred to the web version of this article.)

We investigated whether Nid1-EGFP was incorporated into BMs *in vitro* in mouse ES cell-derived embryoid bodies, in which BMs are formed between the endoderm and epiblast cell layers [24], [25]. Recombinant Nid1-EGFP was added to the culture medium of single cell-suspended mouse ES cells at a concentration of 25 µg/ml. Cells were cultured until they formed aggregates and differentiated into embryoid bodies (Fig. 2A). Green fluorescence of Nid1-EGFP was detected in the BM zone of the embryoid bodies, which colocalized with the immunostaining signal against type IV collagen (Col IV) (Fig. 2B). Time-lapse imaging of individual embryoid bodies revealed that the EGFP-positive area in the BM zone gradually expanded (Fig. 2C), suggesting that recombinant Nid1-EGFP accumulated continuously in the BM zone. Recently, Kadoya et al. demonstrated that recombinant Nid1-EGFP added to an explant culture of the mouse embryonic submandibular gland visualized epithelial BM dynamics in branching morphogenesis [26].

We also constructed cDNA encoding hNid1 fused with a red fluorescent protein, mCherry (Nid1-mCherry), instead of EGFP, and stably transfected it into a mouse ES cell line (Supplementary Fig. 1). Nid1-mCherry fluorescence was not detected in undifferentiated ES cell colonies (data not shown). However, fluorescence became apparent in the BM zone when the cells differentiated into embryoid bodies (Supplementary Fig. 1). In time-lapse observations, mCherry signals in the BM zone gradually thickened when the cyst developed, possibly because of the overproduction of Nid1-mCherry driven by the CAG promoter. The fluorescence observed in the most superficial layer of the embryoid body may indicate that surface visceral endoderm cells uptake the excess Nid1-mCherry by apical endocytosis. This result suggests that the overexpression of Nid1-mCherry had little effect on BM formation and cell differentiation in embryoid bodies.

Based on these *in vitro* observations, we concluded that the fluorescent protein-tagged hNid1, Nid1-EGFP and Nid1-mCherry, could bind to BM proteins and accurately label BMs.

## Generation of Nid1-mCherry reporter mouse lines

**Fig. 3:**
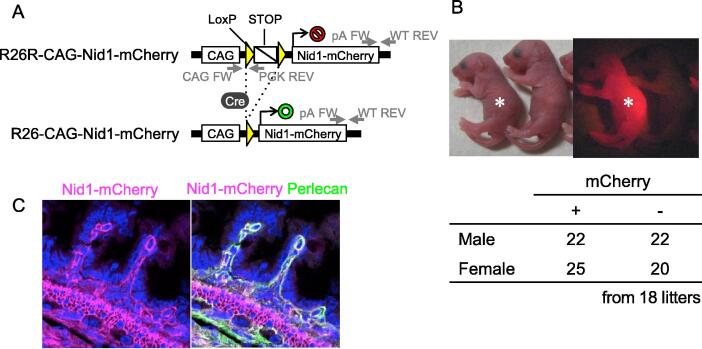
Generation of R26-CAG-Nid1-mCherry mice. A) Scheme of the reporter knock-in at the ROSA26 locus. The Nid1-mCherry is expressed when the STOP cassette is removed by Cre recombinase. CAG, CAG promoter; STOP, a loxP-flanked neomycin-resistant gene with the PGK promoter and SV40 virus triple pA sequences; Nid1-mCherry, hNid1 cDNA with a linker, mCherry, His-tag, and bovine growth hormone pA sequences. Arrows indicate genotyping primers. B) Neonatal pups of R26-CAG-Nid1-mCherry. The pups were obtained from pairs of an R26-CAG-Nid1-mCherry male and a wild-type female. The R26-CAG-Nid1-mCherry pups at P1 showed red fluorescence (asterisk) under 530 nm light, whereas the non-reporter littermates did not. The heterozygous R26-CAG-Nid1-mCherry mice obtained by mating reporter males and wild-type females were born in the expected Mendelian ratio. C) Histological evaluation of R26-CAG-Nid1-mCherry. Tissue sections of the intestine of an R26-CAG-Nid1-mCherry at P1 were immunostained for perlecan. Nid1-mCherry fluorescence (magenta), perlecan immunofluorescence (green), and nuclei (blue) are represented. (For interpretation of the references to color in this figure legend, the reader is referred to the web version of this article.)

To visualize the BMs using the Nid1-mCherry probe *in vivo*, we established a conditional reporter mouse line (R26R-CAG-Nid1-mCherry) expressing Nid1-mCherry under the control of the CAG promoter at the ROSA26 locus via gene targeting in ES cells (Fig. 3A) [22]. A constitutive reporter mouse line (hereafter referred to as R26-CAG-Nid1-mCherry) was generated from R26R-CAG-Nid1-mCherry by removing the loxP-flanked stop sequence. The resulting offspring of R26-CAG-Nid1-mCherry mice were viable and fertile in the heterozygous state. mCherry fluorescence was detected in the skin on postnatal day (P) 1 (Fig. 3B, asterisk). The heterozygous R26-CAG-Nid1-mCherry offspring obtained by mating the reporter males and wild-type females were fertile as their non-reporter littermates and born at the expected Mendelian ratio. Histological inspection of the P1 mice revealed Nid1-mCherry fluorescence in the BM zone of the intestinal epithelium, smooth muscle cells, and blood vessels (Fig. 3C, magenta). Nid1-mCherry fluorescence colocalized with the immunofluorescent signal of perlecan, a BM protein (Fig. 3C, green).

## Distribution of Nid1-mCherry in adult tissues

**Fig. 4:**
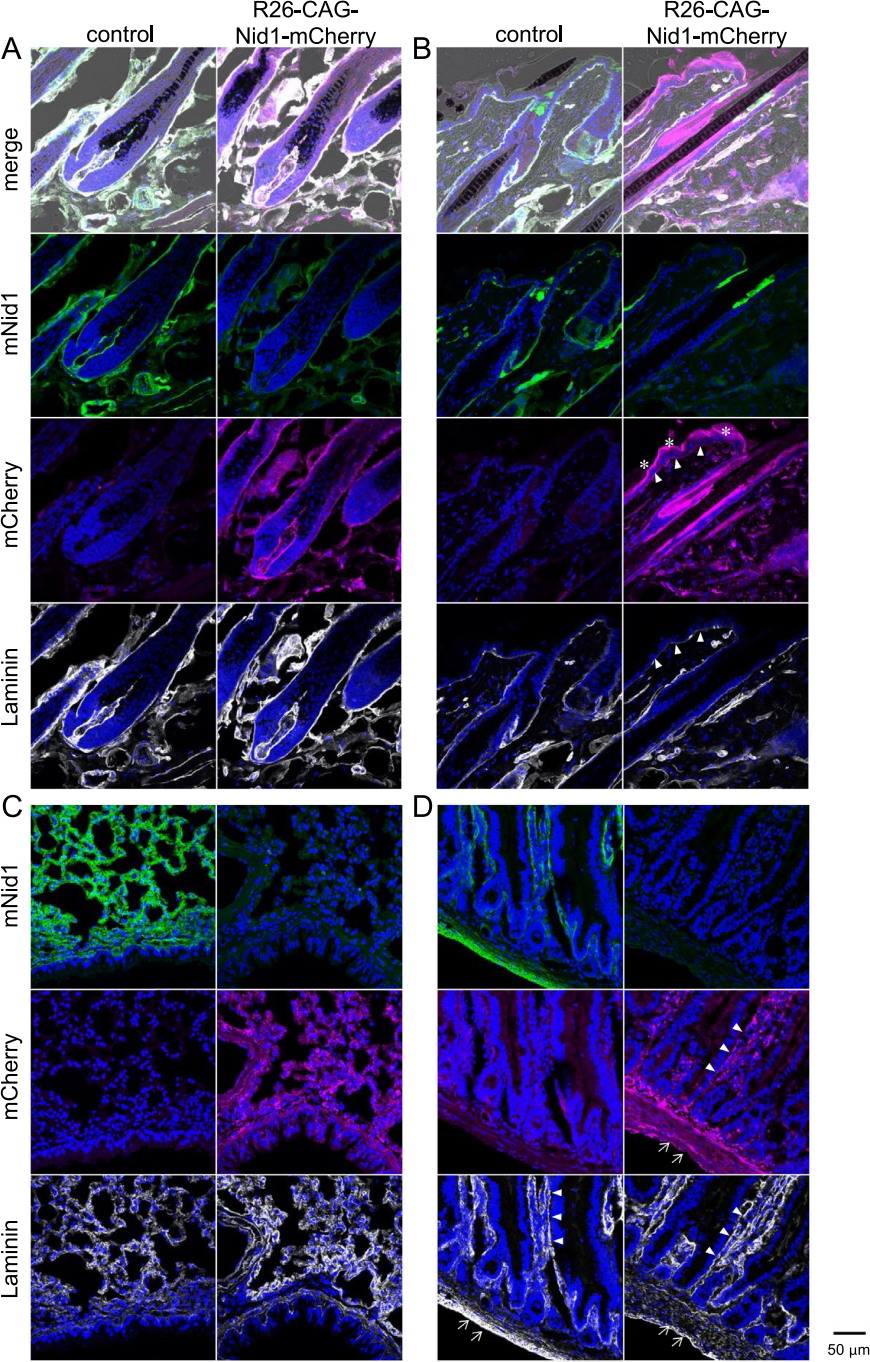
Distribution of Nid1-mCherry in adult tissues.

To investigate Nid1-mCherry expression in adult tissues, representative organs (Table 1) of R26-CAG-Nid1-mCherry mice were examined for the localization of Nid1-mCherry fluorescence and expression of endogenous mouse nidogen-1 (mNid1) and laminin (Fig. 4 and Supplementary Fig. 2). The rat monoclonal antibody against mNid1 (ELM1) reportedly does not cross-react with hNid1 [26].

**Table 1:** Summary of Nid1-mCherry localization in adult tissues.

	Basement membrane labeling	Ectopic signals
Skin	Epidermal BM, including hair follicles, hair bulbs, and dermal papillae	Corneum of the epidermis
Lung	(Almost absent)	Dots in the alveolar stroma
Heart	Cardiac cell BM (Weak and discontinuous in the epicardial and endocardial BMs)	
Aorta	(Not clear because of ectopic signals)	Mesothelium
Skeletal muscle (soleus)	Muscle cell BM	
Cerebral cortex	Pia mater (weak) & capillary endothelial BMs	
Choroid plexus	Epithelial & capillary BMs	
Sciatic nerve	Perineurium & endoneurium (myelin sheath’s BM)	
Retina	Inner limiting membrane & capillary BMs	
Esophagus	Epithelial (weak) & muscle cell BMs	Dots in the subepithelial mucosa (lamina propria)
Stomach	Gastric gland epithelial BM (weak)	
Small intestine	Epithelial (weak) & smooth muscle BMs	
Kidney	Glomerular & renal tubular BMs	A part of the (likely proximal) renal tubular epithelial cell cytoplasm
Uterus	(Almost absent)	Dots in the subepithelial tissue

In the hair bulb region of the skin (Fig. 4A), Nid1-mCherry fluorescence was localized to the BMs surrounding the hair follicles and dermal papillae. These Nid1-mCherry signals colocalized with the immunofluorescence signals of mNid1 and laminin. Notably, the immunofluorescence of mNid1 was weaker in R26-CAG-Nid1-mCherry mice than in their non-reporter littermates (control). Similarly, in the epidermal region of the skin (Fig. 4B), Nid1-mCherry fluorescence was detected in the epidermal BM region (Fig. 4B, arrowheads) and hair follicles and colocalized with the immunofluorescence of laminin, whereas mNid1 immunofluorescence was lower than that in the control. Ectopic non-BM-like Nid1-mCherry signals were observed in the cornified epidermal layer (Fig. 4B, asterisks).

In the lungs (Fig. 4C), the immunostaining signals of mNid1 and laminin in the control tissue indicated that BMs lined the bronchial and alveolar walls; however, the Nid1-mCherry signals were punctate in the stroma and cytoplasm, rather than in the BMs. Despite the non-BM-like localization of Nid1-mCherry fluorescence, mNid1 immunofluorescence in the lungs of reporter mice was lower than that in the controls. Electron microscopy revealed no obvious defects in the alveolar BMs of either R26-CAG-Nid1-mCherry or control mice (Supplementary Fig. 4A and 4B).

In the intestinal microvilli (Fig. 4D), the BMs of the intestinal epithelium and microvasculature were illuminated with the immunofluorescence signals of laminin and mNid1 in the control. Nid1-mCherry fluorescence was detected in epithelial BMs (Fig. 4D, arrowheads) but was ambiguous in microvascular BMs. Instead, punctate red fluorescence signals, which did not overlap with the laminin signals, were observed in the stromal regions of the microvilli. Nid1-mCherry signals were also detected in the BMs of smooth muscle cells in the intestinal wall (Fig. 4D, arrows). Similar to other tissues, the immunofluorescence signals of mNid1 were decreased in the intestines of reporter mice.

Immunostaining of mouse endogenous nidogen-1 (mNid1) and laminin in R26-CAG-Nid1-mCherry mice and their non-reporter littermates (controls) was performed in several adult tissues.

A) Skin: hair follicular bulbs; B) skin: epidermal region (arrowheads, epidermal BM; asterisks, ectopic signals in the cornified epidermal layer); C) lungs; and D) intestinal microvilli (arrowheads, epithelial BM; arrows, smooth muscle cells). Scale bar indicates 50 µm.

The results for other tissues, including the muscles and nervous system, are shown in Supplementary Fig. 2 and summarized in Table 1. In summary, Nid1-mCherry signals were localized to the BMs of cardiac and skeletal muscles, myelin sheaths of peripheral nerve tissue, and capillaries in the brain and retina. However, signals were rarely detected in the BMs of the epicardium, perivascular adipose tissue of the aorta, or pia mater of the brain, whereas they were ectopically strong in the aortic media and part of the renal tubules in the kidney. In most of the tissues examined, mNid1 immunostaining signals were weaker in the R26-CAG-Nid1-mCherry mice than in the control. To investigate whether the absence of Nid1-mCherry fluorescence in the BM region was due to protein degradation, we conducted western blot analysis of tissue extracts from both the lungs and skin, as well as purified recombinant hNid1 and Nid1-mCherry (Supplementary Fig. 3). Recombinant Nid1-mCherry exhibited a higher molecular weight band than hNid1 when subjected to either anti-hNid1 or anti-His-tag antibodies (arrowheads in Supplementary Fig. 3A and 3B), as well as several bands of lower molecular weight, likely indicating degradation products. The anti-His-tag blot revealed several lower molecular weight bands than the anti-hNid1 blot. Specific high-molecular-weight bands were detected in the lungs and skin of R26-CAG-Nid1-mCherry mice, which were absent in the control. Furthermore, the anti-His-tag blot showed low-molecular-weight bands at approximately 20 kDa only in R26-CAG-Nid1-mCherry mice (asterisks in Supplementary Fig. 3B). Because the His-tag is located at the C-terminus of Nid1-mCherry, these results suggest that the Nid1-mCherry protein in mouse tissue is degraded into fragments of multiple sizes, some of which lack the hNid1 region. In addition, blots for mNid1 and β-actin demonstrated a lower amount of endogenous mNid1 in R26-CAG-Nid1-mCherry than in the control (Supplementary Fig. 3C and 3D), suggesting that constitutive expression of Nid1-mCherry eliminates endogenous mNid1 deposition in BMs.

## Visualization of BMs in early embryos

**Fig. 5:**
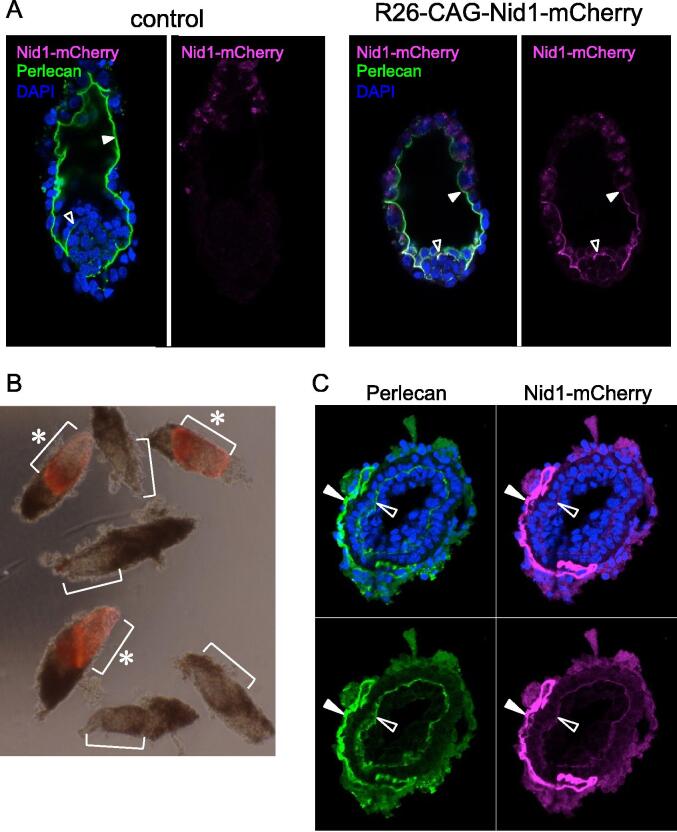
Localization of Nid1-mCherry in early embryos. A) Confocal images of E4.5 blastocysts of control and R26-CAG-Nid1-mCherry immunostained for perlecan. The left panels represent merged images of perlecan (green), Nid1-mCherry (magenta), and nuclei (blue), while the right panels show mCherry single images. B) Whole-mount images of E6.5 egg-cylinder embryos. Brackets indicate the distal embryonic regions. R26-CAG-Nid1-mCherry embryos (asterisks) showed intense red fluorescence in the embryonic regions. C) A transverse section of the R26-CAG-Nid1-mCherry embryos at E6.5. The section was immunostained for perlecan. Perlecan (green) and Nid1-mCherry (magenta) images of the same section are represented with or without nuclei (blue). Reichert’s membrane (solid arrowheads) and the inner embryonic BM (open arrowheads) are indicated. (For interpretation of the references to color in this figure legend, the reader is referred to the web version of this article.)

BM formation is a critical step in early embryogenesis. Because the first BM appeared at the E4.5 blastocyst stage, both pre- and post-implantation embryos were examined **(**Fig. 5). In control blastocysts, BMs visualized by immunostaining for perlecan were located along the luminal surface of the blastocoel (Fig. 5A, solid arrowheads) and between the primitive endoderm and the inner cell mass **(**Fig. 5A, open arrowheads**)**. Nid1-mCherry fluorescence colocalized with perlecan immunofluorescence.

At the E6.5 egg-cylinder stage, the embryos were elongated in shape. The distal part of the R26-CAG-Nid1-mCherry embryos showed red fluorescence **(**Fig. 5B, brackets and asterisks**)**, whereas the control embryos did not **(**Fig. 5B, brackets**)**. In transverse sections of the Nid1-mCherry embryo (Fig. 5C), both the embryonic BM (open arrowheads) and Reichert’s membrane (solid arrowheads) were positive for Nid1-mCherry and perlecan immunostaining. These results indicate that Nid1-mCherry-labeled BMs originated at the beginning of embryogenesis.

## Nid1-mCherry at the endothelial BMs of retinal blood vessels

**Fig. 6:**
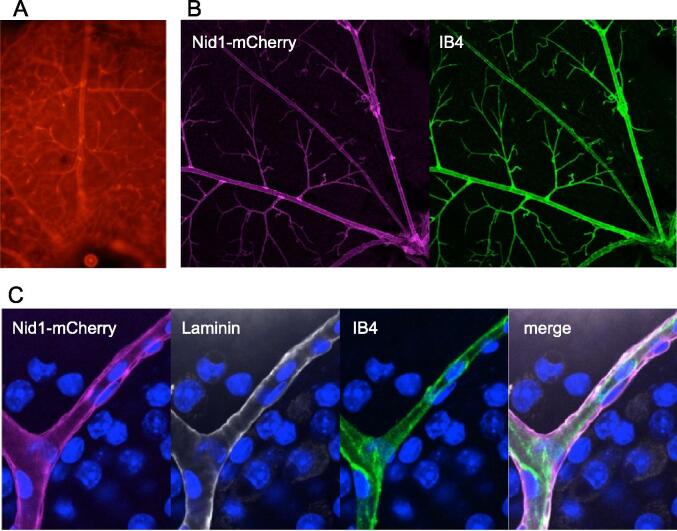
Whole-mount images of retinal vascular BMs of R26-CAG-Nid1-mCherry mice. A) Nid1-mCherry fluorescence in the retina of adult R26-CAG-Nid1-mCherry mice immediately after isolation. The inner surface of the retina was observed using a fluorescence microscope. The branching blood vessel pattern was visible with Nid1-mCherry fluorescence. B) Nid1-mCherry fluorescence in the fixed adult retina. The isolated retina was fixed and stained with IB4. The Nid1-mCherry fluorescence (magenta) was colocalized with the blood vessels visualized with IB4 (green). C) Magnified images of the retinal blood vessels of R26-CAG-Nid1-mCherry mice immunostained for laminin and with IB4. Nid1-mCherry fluorescence and laminin immunofluorescence outline the vessels, while the IB4 signals (green) show the inner endothelial cells. DAPI counterstaining is shown in blue. (For interpretation of the references to color in this figure legend, the reader is referred to the web version of this article.)

To investigate Nid1-mCherry labeling in vascular endothelial BMs in living organs, we observed the blood vessels of retinal explants [27], [28]. The adult retinas of R26-CAG-Nid1-mCherry mice were freshly isolated, mounted on glass-bottomed dishes, and observed using confocal microscopy. Nid-mCherry fluorescence revealed the blood vessels on the inner surface of the retina (Fig. 6A). The retina was fixed and stained with fluorescent-tagged isolectin B4 (IB4) to visualize the endothelial cells. The branching pattern of Nid1-mCherry fluorescence was consistent with that of the IB4-positive vessels (Fig. 6B). In the magnified images, Nid1-mCherry fluorescence was localized in the BM region, which outlined the endothelial cell layer and individual mural cells (Fig. 6C). Electron microscopic observation of the vascular BMs revealed no apparent differences between R26-CAG-Nid1-mCherry and the control in endothelial and pericyte BMs (Supplementary Fig. 4C-4F).

**Fig. 7:**
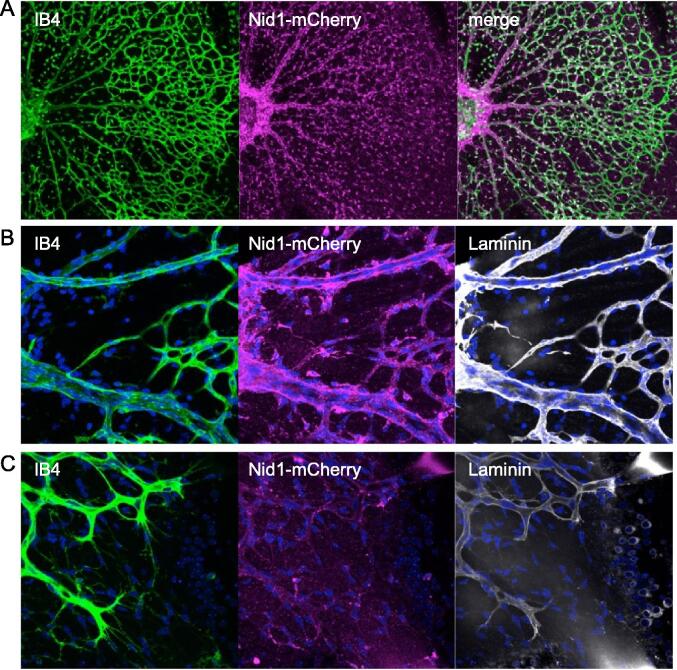
Distribution of Nid1-mCherry in developing retinal vasculature. A) A live image of the developing vascular network in the P5 retina of R26-CAG-Nid1-mCherry mice. The freshly isolated retina was observed in the presence of IB4 to visualize endothelial cells. Unlike adult retinas, Nid1-mCherry signals did not exactly colocalize with IB4. B) Magnified images of the large radial vessels around the optic disc at the center of the P5 retina. Isolated retinas were fixed and immunostained for laminin (gray) and stained with IB4 (green) and DAPI (blue). In large vessels, Nid1-mCherry fluorescence colocalized with that of IB4. C) Magnified images of the growing front of the developing vascular network. IB4 (green) and laminin (gray) indicate endothelial cells and their BMs, respectively, whereas Nid1-mCherry (magenta) did not colocalize with laminin. The nuclei are shown in blue. (For interpretation of the references to color in this figure legend, the reader is referred to the web version of this article.)

**Fig. 8:**
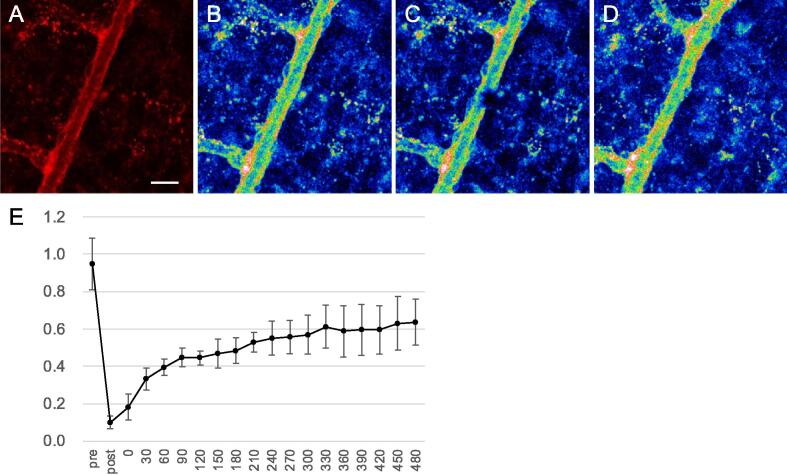
Recovery of Nid1-mCherry fluorescence in the vascular BM after photobleaching. (A) Nid1-mCherry fluorescent images of a vessel with two branches. (B-D) The same vessel is represented in a pseudo-color according to the fluorescence intensities. The vessel before photobleaching (B), immediately after the photobleaching (C), and 12 h later (D) are represented. The retina was incubated for 12 h under time-lapse recording using Leica SP8 equipped with a stage-top incubation chamber. (E) Quantitative analysis of the fluorescence recovery. Relative fluorescence intensities were calculated by a fluorescence value of the bleached area compared against that of the BM on the opposite side of the vessel. The ratios were quantified every 30 min until 8 h after the beginning of the time-lapse recording. Measurements were taken at a minimum of three points from each of the two retina explants derived from different animals. Pre and post; before and after laser irradiation, 0–480; minutes from the beginning of the time-lapse recording. Each value and error bar represent the mean of the fluorescence ratio and the standard deviation, respectively.

Since the retinal vascular network begins to form after birth in mice, the retinas of R26-CAG-Nid1-mCherry mice at P5 were freshly isolated and observed. Endothelial cells were visualized using IB4. Developing vessels spread radially from the central optic disc and formed a capillary plexus (Fig. 7A). Nid1-mCherry signals were distributed along the large radial vessels and surrounding tissue. For further investigation, the retinas were fixed and immunostained for laminin. Nuclei were visualized with 4′,6-diamidino-2-phenylindole (DAPI). In the magnified images of the central large vessels (Fig. 7B), Nid1-mCherry fluorescence outlined the vessel walls and colocalized with laminin immunofluorescence. However, in the growing front of the capillaries at the periphery of the retina, Nid1-mCherry fluorescence was faint and punctate despite the presence of the BM, as indicated by laminin immunostaining signals that outlined the endothelial cells (Fig. 7C). This result suggests that the efficiency of Nid1-mCherry labeling depends on the maturation of vessels or vascular BMs. We performed time-lapse imaging of the two-week-old retina, in which the vascular BMs were clearly labeled with Nid1-mCherry fluorescence (Fig. 8 and Supplementary Movie 2). A vessel outlined with the red fluorescence of Nid1-mCherry in the BM zone (Fig. 8A) is represented in a pseudo-color according to the fluorescence intensities (Fig. 8B). To examine the dynamic changes in the BM, a point on the vessel wall was photobleached using a 561-nm laser (Fig. 8C). The fluorescence at the bleached spot was recovered 14 h later (Fig. 8D). Time-lapse observations revealed a gradual recovery of fluorescence (Supplementary Movie 2), suggesting continuous accumulation of Nid1-mCherry in the BM zone. Relative fluorescence levels were quantified at 30-min intervals during the time-lapse observation, and the recovery ratios were calculated. The recovery ratios were reproducible across multiple points in different retinas, and the average ratio increased for at least 8 h post-irradiation (Fig. 8E).

## Discussion

In this study, we developed hNid1-based probes for the BM imaging of mouse cells and tissues. Both Nid1-EGFP and Nid1-mCherry successfully labeled BMs in mouse ES cell-derived embryoid bodies, an *in vitro* 3D culture model. Recently, Kadoya et al. demonstrated that recombinant Nid1-EGFP visualized the dynamic movements of BMs associated with epithelial morphogenesis of the developing submandibular gland in mouse embryos [26]. These results suggest that Nid1-EGFP can be used to visualize BM dynamics in various systems. As an *in vivo* BM-imaging model, R26-CAG-Nid1-mCherry mice showed fluorescently labeled BMs in multiple tissues and organs and the vascular BMs of living retinal explants. To the best of our knowledge, this is the first demonstration of a genetically engineered vertebrate BM labeling model.

Because the formation and maintenance of BMs are critical for tissue organization and are closely related to pathological changes, the visualization of BM dynamics in mammalian tissues is required to elucidate the underlying mechanism by which BM assembly is regulated. Although genetic tagging of BM-specific proteins has been achieved in Drosophila and Caenorhabditis elegans [7], [11], [12], [13], genetic modification of BM-specific proteins in mammalian cells often disturbs their expression, function, or both [29], [30]. Shaw et al. reported that genetic tagging of the laminin β1 subunit on its C-terminus with a photoconvertible fluorescent protein prevented the subunit from being secreted from the human lung carcinoma cell line A549, even though it was translated in the cells [31]. We also attempted to tag the laminin α5 subunit with EGFP at the N-terminus. However, it was not secreted by the 293F cells (data not shown). Therefore, we chose nidogen-1 as the probe because it is a monomeric protein with a binding affinity for other BM-specific proteins. Both Nid1-EGFP and Nid1-mCherry were secreted into the conditioned media when transfected into mammalian cells and retained their BM-binding abilities. Because Nid1-mCherry appeared to be secreted more efficiently than Nid1-EGFP (data not shown), we generated BM-imaging transgenic mice with Nid1-mCherry.

R26-CAG-Nid1-mCherry mice were viable and fertile, and no macroscopic or histological abnormalities were observed, suggesting that the overexpression of Nid1-mCherry in mice did not disturb the critical function of BMs. Immunofluorescence and western blot analyses revealed that the localization of endogenous mNid1 in BMs was decreased in multiple organs of R26-CAG-Nid1-mCherry mice. These results suggest that the overexpression of the Nid1-mCherry protein may compete with endogenous mNid1 for integration or accumulation in BMs. Although recombinant Nid1-mCherry was derived from human nidogen-1, it shared 85% amino acid identity with mouse nidogen-1. Overexpression of Nid1-mCherry driven by the CAG promoter overrides mouse nidogen-1 in terms of BM incorporation efficiency.

Although there is a broad potential to study BM dynamics using R26-CAG-Nid1-mCherry, it has some limitations as an *in vivo* BM imaging model. In organs such as the lungs and uterus, Nid1-mCherry fluorescence is scattered in the stromal tissue rather than in the BMs. Strong ectopic signals in some parts of the renal tubules may disrupt the observation of tubular BMs. Western blot analysis suggested the partial degradation of Nid1-mCherry in mouse tissues, partially explaining the inconsistency between mCherry fluorescence and BM regions. In addition, because Nid1-mCherry expression is driven by a ubiquitous CAG promoter, high mechanical or physiological stress may result in unregulated hyperproduction of Nid1-mCherry, causing ectopic high fluorescent signals in the epidermal cornified layer or tunica media of the aorta. In the developing retinal vascular network, Nid1-mCherry signals were observed in the vascular BMs of proximal vessels but not in the distal capillary plexus, where the growing tips of endothelial cells lead to angiogenesis [28]. Although the degree of BM labeling by Nid1-mCherry varies among tissues/organs and developmental processes, further investigation may provide insights into BM assembly and dynamics in mammals.

Genetic labeling of BMs in living organisms using fluorescent proteins stands out for the histological resolution and analysis of molecular dynamics. For example, Keeley et al. reported the dynamic movements of BM components within the BM zone by comprehensively tagging each component in Caenorhabditis elegans [7]. In addition, local and limited degradation of BM components by matrix metalloproteinases is critical for developing processes such as branching morphogenesis [14] and angiogenesis [32], [33]. Monitoring the dynamics of fluorescently labeled BM proteins will provide new insights into the mechanisms of BM assembly, degradation, and maintenance in the context of interactions with epithelial and endothelial cells during organogenesis, tissue homeostasis, and pathogenesis. Additionally, the R26R-CAG-Nid1-mCherry line was designed to express Nid1-mCherry in a Cre-dependent manner, allowing for tissue- and cell-type-specific studies of BM dynamics. This study provides a model for further *in vivo* studies of BM dynamics. It also provides the advantage of visualizing organogenesis *in vitro* using stem cell-derived organoids.

## Materials and methods

### Construction and expression of recombinant hNid1, nid1-EGFP, and Nid1-mCherry

The expression vector for hNid1 was prepared as described previously [34]. For Nid1-EGFP or Nid1-mCherry expression, the termination codon of hNid1 cDNA was substituted with a cDNA encoding EGFP and mCherry, respectively, with a short linker sequence encoding four amino acids at the 5′-flanked region and a sequence encoding six histidine residues (His-tag) and a termination codon at the 3′-flanked region. EGFP and mCherry cDNA were derived from pEGFP-N1 and pmCherry vectors, respectively (Clontech Takara Bio, Otsu, Japan). Recombinant hNid1 and Nid1-EGFP were expressed using the FreeStyle 293 Expression System (Thermo Fisher Scientific, Waltham, MA, USA) according to the manufacturer's instructions and affinity-purified from the conditioned media using a Ni-NTA agarose column as described previously [34], [35]. Purified proteins were subjected to SDS-PAGE and immunoblotting using an anti-His-tag monoclonal antibody (Qiagen, Hilden, Germany).

### Solid-phase binding assay

The binding activities of recombinant hNid1 and Nid1-EGFP to other ECM proteins were quantified using solid-phase binding assays, as described previously [34]. Briefly, 96-well plates (MaxiSorp^TM^; Nunc Thermo Fisher Scientific) were coated with the indicated ECM proteins at 10 nM overnight at 4 °C, followed by washing with Tris-buffered saline (TBS) and blocking with 3% bovine serum albumin (BSA). The plates were then incubated with 10 nM recombinant hNid1 or Nid1-EGFP for 1 h at room temperature. After three washes with TBS containing 0.05% Tween-20, the bound Nid1-EGFP was detected using an anti-human nidogen-1/entactin antibody (MAB2570; R&D Systems, Minneapolis, MN) and Peroxidase AffiniPure F(ab')₂ Fragment Rabbit Anti-Mouse IgG (H + L) (315–036-003; Jackson ImmunoResearch, West Grove, PA). The amount of horseradish peroxidase-conjugated antibody bound to the plate was quantified by measuring the absorbance at 490 nm after incubation with *o*-phenylenediamine (FUJIFILM Wako Pure Chemical, Osaka, Japan). Human type IV collagen (Col IV) was purchased (354245; Becton Dickinson Franklin Lakes, NJ, USA). Laminin-111 (LN111) and perlecan (Perl) were recombinantly expressed and purified as previously described [34], [36]. Fibronectin was purified from human plasma as previously described [37].

For the two-step binding assay, a 96-well plate (MaxiSorp^TM^; Nunc Thermo Fisher Scientific) was coated with human type IV collagen (C5533, Sigma-Aldrich, St. Louis, MO) at 100 nM overnight at 4 °C. The coated plate was overlayed with recombinant Nid1-EGFP at 10, 30, or 100 nM for 1 h at room temperature, washed with TBST, and incubated with 10 nM recombinant human laminin with a FLAG-tag [38] or BSA for 1 h at room temperature. The amount of laminin captured on the plate was detected using an anti-FLAG antibody and HRP-conjugated secondary antibody and quantified by measuring the absorbance at 490 nm after incubation with *o*-phenylenediamine (FUJIFILM Wako Pure Chemical).

### Mouse ES cell culture and differentiation of embryoid bodies

The mouse ES cell line, EB5 [39], was maintained as previously described. Briefly, undifferentiated cells were maintained on gelatin-coated dishes without feeder cells in Glasgow minimal essential medium (GMEM; Sigma-Aldrich, St. Louis, MO) supplemented with 10% fetal calf serum, 10% knockout serum replacement (Invitrogen Thermo Fisher Scientific), 1 mM sodium pyruvate, 0.1 mM 2-mercaptoethanol, and 1,000 U/ml leukemia inhibitory factor (LIF; ESGRO; Merck Millipore, Burlington, MA).

Embryoid bodies were differentiated as previously described [24]. Briefly, ES cells were dissociated and cultured under non-adhesive conditions at a density of 2 × 10^5^ cells/ml without LIF. The cells were incubated for seven days to form aggregates and differentiate autonomically.

### Time-lapse imaging of embryoid bodies

Embryoid bodies differentiated in the presence of recombinant Nid1-EGFP for 6–7 days were individually captured in small agar wells in a glass-bottomed dish (AGC Techno Glass, Shizuoka, Japan). Time-lapse images of the BMs in the embryoid bodies were recorded at 20 min intervals using an LSM5 PASCAL confocal laser scanning microscope (Zeiss) with a culture chamber system (INUBG2-WELS; Tokai Hit, Shizuoka, Japan).

### Generation of R26-CAG-Nid1-mCherry mouse line

The conditional ROSA26 reporter mouse line R26R-CAG-Nid1-mCherry (Accession No. CDB0311K: https://large.riken.jp/distribution/reporter-mouse.html) was established as described previously [22]. To generate the ROSA26 targeting vector, the Nid1-mCherry cDNA was cloned into the pENTR2B vector (pENTR2B-Nid1-mCherry) and inserted into the pROSA26-CAG-STOP-DEST vector using the Gateway system (Thermo Fisher Scientific). pROSA26-CAG-STOP-DEST was modified from pROSA26-STOP-DEST using the CAG promoter (CAG) [40]. The targeting vector was introduced into the C57BL/6N mouse ES cells (HK3i) [41]. After screening the recombinant ES cells, chimeric mice were produced, and germline transmission was confirmed through the next generation. The R26-CAG-Nid1-mCherry mouse line (accession No. CDB0320K: https://large.riken.jp/distribution/reporter-mouse.html) constitutively expressing Nid1-mCherry was generated by crossing R26R-CAG-Nid1-mCherry with EIIa-Cre mice [42]. The offspring were genotyped using the following primers: WT allele (5′-TCC CTC GTG ATC TGC AAC TCC AGT C-3′ (WT FW) and 5′-AAC CCC AGA TGA CTA CCT ATC CTC C-3′ (WT REV), 217 bp), R26-CAG-Nid1-mCherry allele (5′-TCC TGG GCA ACG TGC TGG-3′ (CAG FW) and 5′-TGT GGA ATG TGT GCG AGG CCA GAG G-3′ (PGK REV), 217 bp), and R26R/R26-CAG-CAG-Nid1-mCherry alleles (5′-GGG GGA GGA TTG GGA AGA CAA TAG C-3′ (pA FW) and WT REV, 297 bp). The R26-CAG-Nid1-mCherry line was maintained by mating a heterozygous male with a wild-type C57BL/6J female. All histological analyses and live observations were performed on heterozygous animals that showed clear Nid1-mCherry fluorescence.

All animal experiments were approved by the Institutional Animal Care and Use Committees of RIKEN Kobe Branch and the Institutional Review Board of Osaka Medical and Pharmaceutical University and performed in accordance with the RIKEN animal experimentation guidelines and procedures outlined in the Guide for the Animal Care and Use of Laboratory Animals of Osaka Medical and Pharmaceutical University.

### Preparation of tissue sections for immunofluorescence

To collect tissues and embryos, mice were sacrificed by cervical dislocation under deep anesthesia with isoflurane inhalation. The tissues and embryos of R26-CAG-Nid1-mCherry and non-reporter littermates were embedded in Tissue-Tek® O.C.T. Compound (Sakura Finetek Japan), frozen with liquid nitrogen, and sectioned at 10-µm thickness using a Leica CM3050S (Leica, Wetzlar, Germany). Cryosections were fixed with 4% paraformaldehyde (PFA) for 15 min, blocked with PBS containing 2% BSA for 30 min at room temperature, and incubated with primary antibodies overnight at 4 °C. The sections were probed with secondary antibodies for 1 h at room temperature and counterstained with DAPI. For whole-mount immunostaining, the dissected embryos were fixed in 4% PFA for 30 min at room temperature, permeabilized in PBS containing 0.1% Triton X-100 and 0.1% Tween-20 (PBST), and blocked in PBST containing 2% BSA for 1 h at 4 °C. The embryos were incubated with primary and secondary antibodies overnight at 4 °C. Immunofluorescence images were obtained using a TCS SP5 laser scanning microscope (Leica).

### Western blot of mouse tissues

Mice were sacrificed by cervical dislocation under deep anesthesia with isoflurane inhalation, and their lungs and skin were collected. Tissues were homogenized in an extraction buffer with protease inhibitors (RIPA lysis buffer system, Nacalai Tesque, Kyoto, Japan) and incubated overnight at 4 °C. The homogenates were centrifuged, and the supernatants were subjected to SDS-PAGE under reducing conditions. Purified recombinant hNId1 and Nid1-EGFP (20 ng/lane) were also analyzed. The separated proteins were transferred onto a PVDF membrane (Immobilon-P; Millipore). After blocking with 2% BSA in TBST, the membrane was incubated with primary antibodies against hNid1 (MAB2570, R&D Systems), His-tag (Qiagen), mNid1 (ELM1; Santa Cruz Biotechnology, Dallas, TX), or β-actin (13E5, Cell Signaling Technology, Danvers, MA) overnight at 4 °C. After incubation with HRP-conjugated secondary antibodies for 1 h at 4 °C, the products were detected with Amersham™ ECL™ Prime Western Blotting Detection Reagent (Cytiva, Tokyo, Japan) using an imaging system (ChemiDoc XRS+, Bio-Rad, Hercules, CA).

### Preparation and live observation of mouse retina

Mice were sacrificed by cervical dislocation under deep anesthesia with isoflurane inhalation for adults and hypothermia for pups. Retinas were isolated according to the method described by Sawamiphak et al. [27]. The eyes of R26-CAG-Nid1-mCherry mice were removed, and the retinas were detached from other ocular tissues and flattened for observation. For whole-mount immunostaining, retinas were fixed with 4% PFA and probed with primary and secondary antibodies as described above. Alexa Fluor 488-conjugated IB4 (I21411; Thermo Fisher Scientific) was used to visualize the endothelial cells. The specimens were observed under a TCS SP8 laser-scanning microscope (Leica). Live imaging of the 2-week-old retina was performed using a TCS SP8 (Leica) equipped with a stage-top incubator system (CK-150A; Blast Inc., Kanagawa, Japan). Time-lapse images were recorded at 10 min intervals. Photobleaching was performed by single irradiation with a 532-nm laser for 5 s. To quantify Nid1-mCherry fluorescence, serial images taken before and immediately after irradiation, as well as every 30 min during time-lapse observation, were analyzed using Fiji/ImageJ [43]. The relative fluorescence intensities were calculated as the ratio of the fluorescent values of the bleached area to that of the BM on the opposite side of the vessel. Measurements were obtained at a minimum of three points from each of the two retinal explants derived from different animals. Quantification was performed on measurements collected until 8 h post-irradiation, as not all time-lapse recordings were successful after 8 h.

### Antibodies and reagents

The primary antibodies used in this study were as follows: anti-penta-His mouse monoclonal antibody (Qiagen), anti-mammalian type IV collagen rabbit polyclonal antibody (Rockland Immunochemicals, Pottstown, PA), anti-heparan sulfate proteoglycan (perlecan) rat monoclonal antibody (A7L6; Millipore), anti-EHS laminin rabbit polyclonal antibody (L9393; Sigma-Aldrich), and anti-mouse nidogen-1 rat monoclonal antibody (ELM1; Santa Cruz Biotechnology, Dallas, Tx). Horseradish peroxidase-conjugated donkey anti-mouse IgG (Jackson ImmunoResearch) was used as a secondary antibody for western blotting. Alexa Fluor™ 488-conjugated goat anti-rat IgG, Alexa Fluor™ 594-conjugated goat anti-rat IgG, Alexa Fluor™ 594-conjugated donkey anti-rabbit IgG, and Alexa Fluor™ 680-conjugated donkey anti-rabbit IgG (Thermo Fisher Scientific) were used for immunofluorescence histochemistry and Alexa Fluor ™ 488-conjugated IB4 (Thermo Fisher Scientific) for visualization of endothelial cells. DAPI or Hoechst33342 was used for nuclear counterstaining.

### Electron microscopy

Adult mice were anesthetized with isoflurane inhalation and mixed anesthetic agents, 0.75 mg/kg Domitor (NIPPON ZENYAKU KOGYO, Japan), 4 mg/kg Midazolam (SANDOZ, Japan), and 5 mg/kg Vetorphale (Meiji Animal Health, Japan), and perfused with 4% paraformaldehyde and 2.5% glutaraldehyde in 0.1 M phosphate buffer (pH 7.4). The lungs and retinas were dissected and post-fixed in 1% OsO_4_. After dehydration with a dilution series of ethanol, the tissues were infiltrated with an epoxy resin (Nisshin-EM, Japan), mounted, polymerized by incubation at 60 °C for two days, and sectioned using an ultramicrotome (RMC PTX, RMC, Japan). Ultrathin sections (approximately 80 nm) were stained with lead citrate and uranyl acetate and examined under a transmission electron microscope (HT7800; HITACHI, Japan).

### CRediT authorship contribution statement

**Sugiko Futaki:** Conceptualization, Investigation, Writing – original draft, Project administration, Funding acquisition. **Ayano Horimoto:** Investigation. **Chisei Shimono:** Investigation. **Naoko Norioka:** Investigation. **Yukimasa Taniguchi:** Investigation. **Hitomi Hamaoka:** Investigation. **Mari Kaneko:** Resources. **Mayo Shigeta:** Resources. **Takaya Abe:** Resources. **Kiyotoshi Sekiguchi:** Supervision, Writing – review & editing. **Yoichi Kondo:** Supervision, Writing – review & editing.

## Declaration of Competing Interest

The authors declare the following financial interests/personal relationships which may be considered as potential competing interests: K.S. is a cofounder and shareholder of Matrixome, Inc.

## Data Availability

Data will be made available on request.

## Appendix A Supplementary data

Supplementary video 5 A time-lapse movie of the embryoid body shown in Figure 2C. A series of EGFP fluorescence images (left) and those merged with phase-contrast images (right). On day 7 of differentiation, the embryoid body was observed using a confocal microscope for 24 h at 20 min intervals.

Supplementary video 6 Time-lapse observation of 2-week-old retinas after photobleaching at a spot of the vascular BM. The isolated retina was cultured under observation by confocal microscopy for 12 h and recorded at 10 min intervals.
