# Supplementary material for: Visualization of basement membranes by a nidogen-based fluorescent reporter in mice
Source: Matrix Biol Plus. 2023 Apr 8;18:100133. doi: 10.1016/j.mbplus.2023.100133 (PMC10149278; doi:10.1016/j.mbplus.2023.100133)
Supplement: Supplementary data 1 [file mmc1.pdf]

Supplementary Figure 1

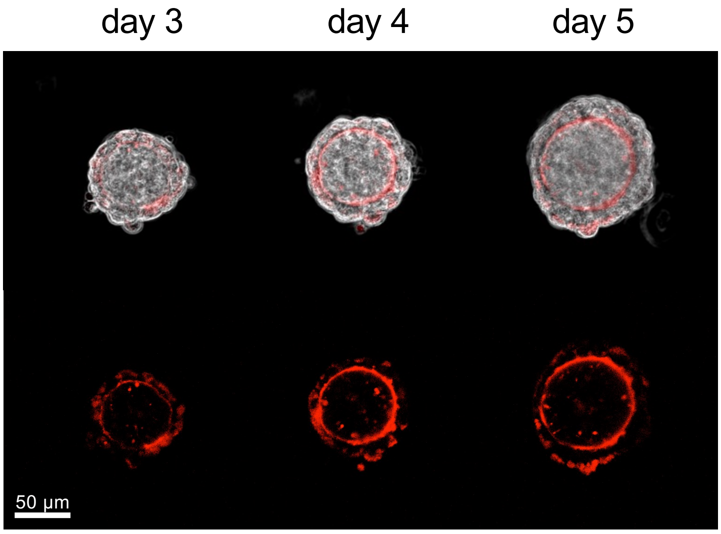

Supplementary Figure 1. Nid1-mCherry labeling of the BM in an embryoid body Time-lapse images of a single embryoid body derived from Nid1-mCherry expressing ES cells. Upper panels show merged images of Nid1-mCherry fluorescence and phase contrast, whereas lower panels show fluorescence alone. Nid1-mCherry fluorescence appeared and thickened in the BM zone as the embryoid bodies differentiated.
