# Supplementary material for: Visualization of basement membranes by a nidogen-based fluorescent reporter in mice
Source: Matrix Biol Plus. 2023 Apr 8;18:100133. doi: 10.1016/j.mbplus.2023.100133 (PMC10149278; doi:10.1016/j.mbplus.2023.100133)
Supplement: Supplementary data 2 [file mmc2.pdf]

Supplementary Figure 2

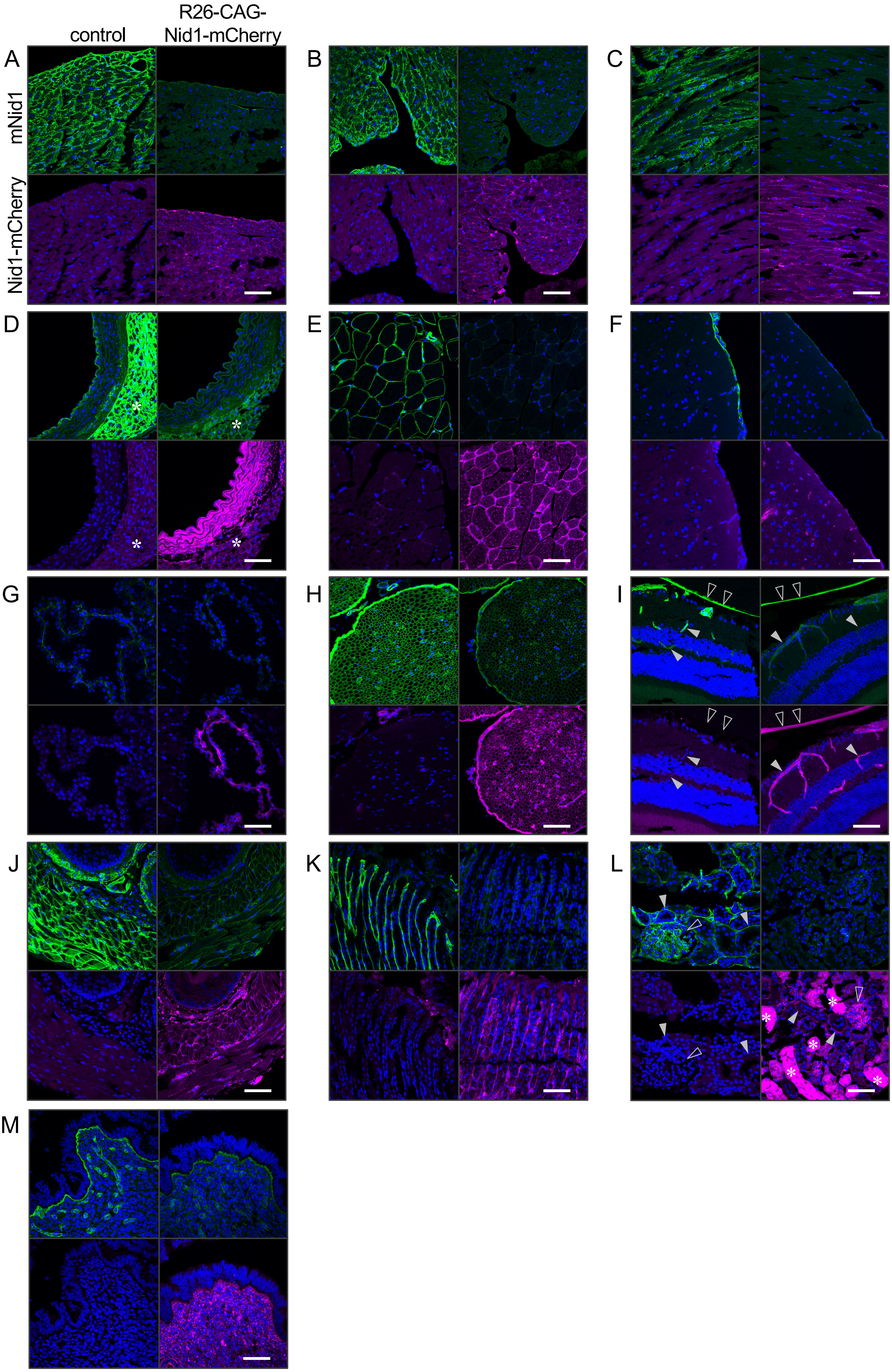

Supplementary Figure 2. Localization of Nid1-mCherry in adult tissues (Supplement to Figure 4)

A) Epicardium, B) endocardium, C) myocardium, D) aorta and adjacent adipose tissue (asterisks), E) skeletal muscle of soleus, F) pia mater of the cerebral cortex and capillaries, G) choroid plexus of lateral ventricle, H) sciatic nerve, I) retina, inner limiting membrane (open arrowheads) and capillaries (solid arrowheads), J) esophagus wall, K) stomach, L) kidney, glomerulus (open arrowheads), renal tubules (solid arrowheads), and renal tubules with ectopic Nid1-mCherry signal (asterisks), and M) uterine endometrium. Left and right columns of each panel show control and R26-CAG-Nid1-mCherry mice, respectively. Green: mNid1, magenta: Nid1-mCherry, blue: nuclei. Scale bars: 50 μm.
