# Supplementary material for: Visualization of basement membranes by a nidogen-based fluorescent reporter in mice
Source: Matrix Biol Plus. 2023 Apr 8;18:100133. doi: 10.1016/j.mbplus.2023.100133 (PMC10149278; doi:10.1016/j.mbplus.2023.100133)
Supplement: Supplementary data 3 [file mmc3.pdf]

Supplementary Figure 3

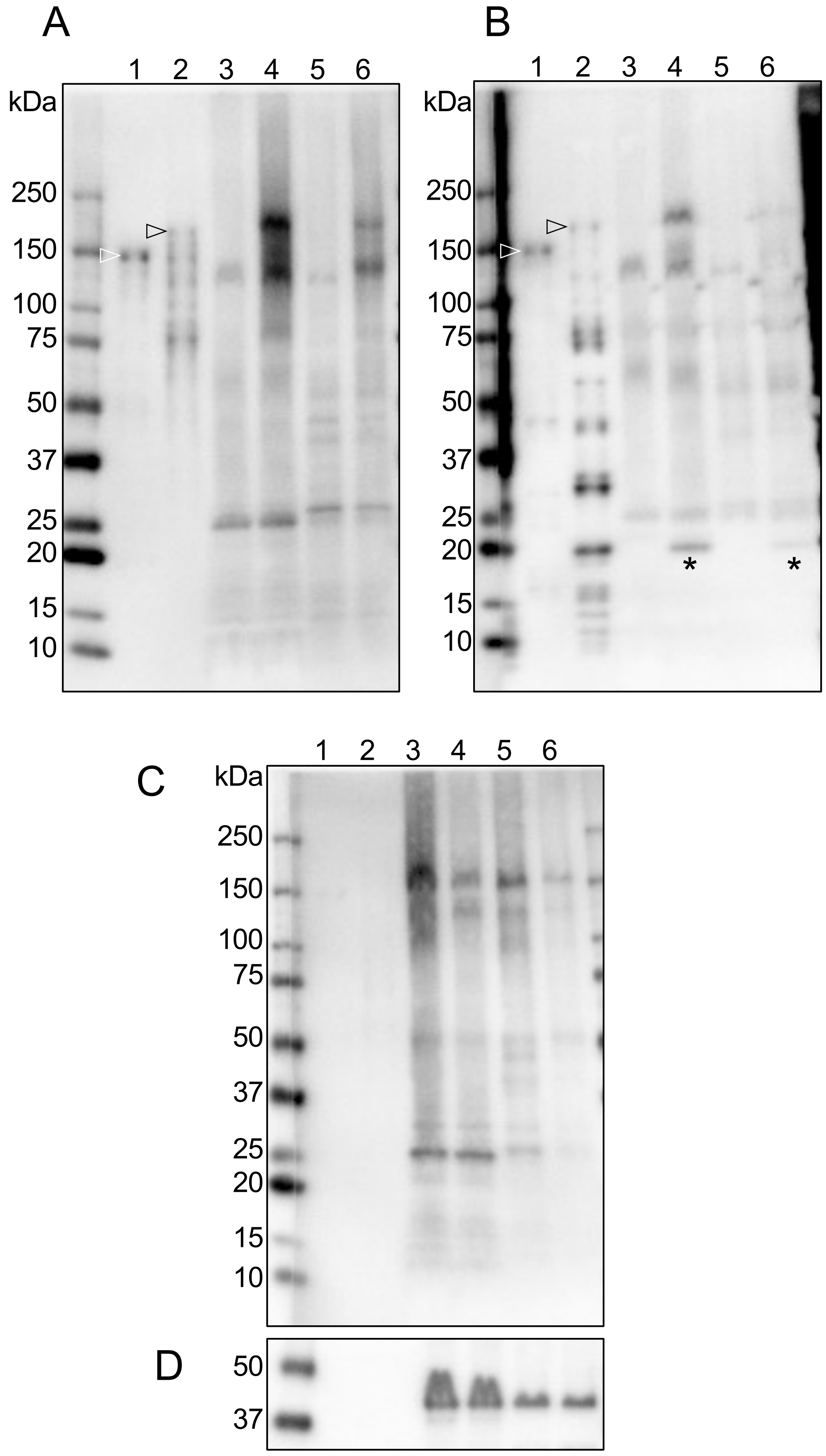

Supplementary Figure 3. Western blot analysis of the lung and skin of control and Nid1-mCherry reporter mice

Recombinant hNid1, Nid1-mCherry, and tissue extracts from the lung and skin of control and R26-CAG-Nid1-mCherry mice were subjected to SDS-PAGE under a reducing condition and western blot was performed with the following antibody incubations: anti-hNid1 (A), anti-His-tag (B), anti-mNid1 (C), and anti-β-actin (D). Lane 1: recombinant hNid1; 2: recombinant Nid1-mCherry; 3 and 4: lungs of the control and R26-CAG-Nid1-mCherry, respectively; 5 and 6: skin of the control and R26-CAG-Nid1-mCherry, respectively.
