# Supplementary material for: Visualization of basement membranes by a nidogen-based fluorescent reporter in mice
Source: Matrix Biol Plus. 2023 Apr 8;18:100133. doi: 10.1016/j.mbplus.2023.100133 (PMC10149278; doi:10.1016/j.mbplus.2023.100133)
Supplement: Supplementary data 4 [file mmc4.pdf]

# Supplementary Figure 4

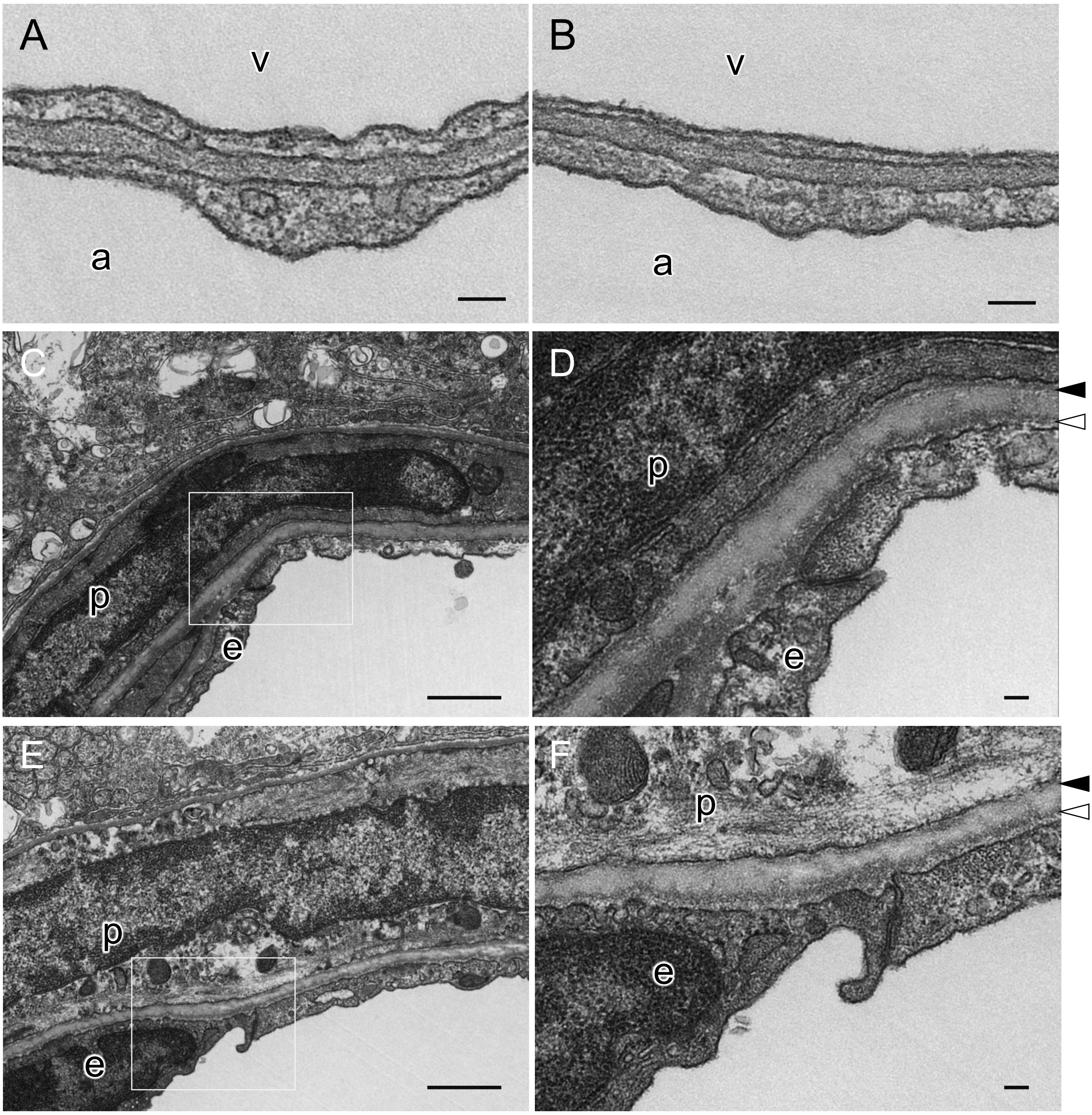


**Supplementary Figure 4. Electron microscopy of the lung and retinal blood vessels**

A and B) Lung alveolar/endothelial BMs of the control (A) and R26-CAG-Nid1-mCherry (B). (C-F) Retinal vascular BMs of the control (C, D) and R26-CAG-Nid1-mCherry (E, F). D and F show magnified images of the rectangles in C and E, respectively. v; vascular lumen, a; air space, p; pericytes, e; endothelial cells. Solid arrowheads; pericyte BMs, open arrowheads, endothelial BMs. Scale bars: 100 nm (A, B, D, F) and 1 µm (C, E).
